# Frontiers in movement disorder therapeutics: A new era in neurology

**DOI:** 10.1016/j.prdoa.2024.100263

**Published:** 2024-07-18

**Authors:** Roongroj Bhidayasiri, Daniel D. Truong

**Affiliations:** Chulalongkorn Centre of Excellence for Parkinson’s Disease & Related Disorders, Department of Medicine, Faculty of Medicine, Chulalongkorn University and King Chulalongkorn Memorial Hospital, Thai Red Cross Society, Bangkok 10330, Thailand; The Academy of Science, The Royal Society of Thailand, Bangkok 10330, Thailand; The Parkinson’s and Movement Disorder Institute, Orange Coast Memorial Medical Center, Fountain Valley, CA 92708, USA

**Keywords:** Movement disorder therapeutics, Treatment landscape Challenges, Personalised medicine

Editorial

Neurology, traditionally focused on diagnostics, has experienced a profound evolution in recent years [1]. With the advancements in medicine and technology, neurologists have transcended mere identification of neurological disorders to spearhead their management and treatment. This paradigm shift is notably evident in the field of movement disorders, where a substantial increase in the affected population is projected between 2010 and 2050 [2]. This trajectory is exemplified by Parkinson’s disease (PD), standing out as the fastest-growing global neurological disorder in terms of disability, mortality, and age-standardised prevalence [3]. With the rapid transition into an ageing society propelled by medical advancements, it is becoming increasingly evident that most reported estimates on the prevalence of movement disorders are conservative. In fact, some experts are now asserting that we are amidst a Parkinson pandemic era [4]. As global citizens, it is paramount that we redefine the landscape of patient care by exploring, expanding, and innovating new therapeutic interventions for a spectrum of movement disorders. While significant strides have been made in addressing conditions like PD, there remain other movement disorders that pose ongoing complexities.

This editorial, featured in a special issue of the Clinical Parkinsonism and Related Disorders journal titled 'Frontiers in Movement Disorder Therapeutics,' aims to contribute to the dawn of a new era in neurology by addressing a range of unfulfilled therapeutic requirements and positively influencing the trajectory of this discipline, serving as a valuable resource in improving the quality of care and outcomes for people with movement disorders. This compilation features seven articles authored by 26 contributors from around the globe. The series on hyperkinetic disorders commences with the exploration of botulinum toxin (BoNT) therapy in oromandibular dystonia (OMD) [5]. The intricate involvement of muscles in OMD necessitates careful patient selection, precise muscle targeting techniques, and careful titration of BoNT dosage. The authors emphasised the need for clinical recognition of OMD subtypes, patient-centric evaluation, and a muscle-targeting approach to dosing to minimise adverse events in the delicate orofacial region. Future studies are encouraged to establish the efficacy of BoNT in different subtypes of OMD through controlled clinical trials to expand therapeutic approaches for this challenging disorder. The second article addresses the treatment of various startle and related disorders, ranging from hyperekplexia and startle-induced disorders to culture-specific syndromes, and functional startle syndromes [6]. It emphasises the multidisciplinary approach required for treatment, including pharmacotherapy and a variety of non-pharmacological interventions like behavioural therapy, physical therapy, and occupational therapy. The review concludes by highlighting the importance of increasing awareness amongst healthcare professionals in addressing the treatment of startle and related disorders and calls for further research in this field.

The third article provides an in-depth review of infection-related movement disorders and their management, covering a wide range of infections that can lead to secondary movement disorders [7]. It highlights the diverse presentations, underlying pathophysiology, common infections associated with movement disorders, and the essential approach to diagnosing and treating these conditions. This article effectively underscores the significance of early diagnosis, suitable treatment, and the use of immunomodulators in specific cases. It discusses a range of infections including tuberculosis, neurocysticercosis, HIV, and COVID-19, among others, which can result in movement disorders such as chorea, ataxia, myoclonus, parkinsonism, and various other manifestations. The final article in the series on hyperkinetic disorders tackles the management challenges posed by ataxias, which often lack definitive cures or disease-modifying therapies across genetic, acquired, or idiopathic origins [8]. While treatments can alleviate certain symptoms and underlying causes to a degree, they are unable to reverse existing damage. The narrative underscores the pivotal role of healthcare professionals in enhancing the quality of life (QoL) for individuals with ataxias through symptom management, provision of information and support, addressing practical issues like insurance and disability, and active involvement in ongoing research aimed at exploring novel therapeutic approaches such as gene therapies and rehabilitation interventions. Common queries from individuals with ataxias about the condition, its aetiology, progression, available treatments, recent research developments, and potential risks to offspring are addressed. The article also lays out diagnostic methods, available treatment options, advancements in ataxia therapy research, and the unpredictable nature of disease progression, stressing the importance of multidisciplinary care and continuous progress to enhance patient outcomes and QoL.

The series on hypokinetic disorders begins with a systematic review delving into the treatment options for axial postural abnormalities in parkinsonian disorders [9]. An array of interventions, from pharmacological treatments to BoNT injections and deep brain stimulation (DBS), have undergone scrutiny for their effectiveness in addressing postural maladies like camptocormia, Pisa syndrome, antecollis, and related conditions. Research has indicated positive outcomes in managing postural abnormalities with interventions such as levodopa, muscle-specific BoNT injections, and DBS targeting the subthalamic nucleus and global pallidus interna. However, results have varied across interventions and cases, underscoring the necessity for tailored approaches contingent on the nature and severity of postural abnormalities in parkinsonian disorders. The second article explores self-treatment methods for freezing of gait (FOG) in individuals with PD using silicone pads to stimulate plantar acupoints with Thai acupressure [10]. This study conducted a trial comparing an active-treatment group utilising silicone pads to a sham-treatment group. Findings revealed that the active treatment led to significant enhancements in stride length, gait velocity, and cadence, while also reducing FOG episodes, FOG duration, %FOG, and double-support time compared to the sham treatment. The research postulated that acupressure with silicone pads represents a secure, non-invasive approach capable of enhancing gait and mitigating FOG in individuals with PD, potentially providing a straightforward self-treatment avenue beyond traditional clinical environments. The final article on managing nocturnal motor disorders offers a comprehensive exploration of the spectrum of nocturnal movement disorders in individuals with PD [11]. It delves into the complexities of nocturnal and sleep-related motor symptoms, providing insights into different manifestations ranging from moving too little to moving too much. It highlights the challenges in diagnosing these disorders due to the need for sophisticated tools like video polysomnography. The review covers various treatment strategies, including non-pharmacological methods such as lifestyle interventions, environmental adjustments, pharmacological therapies (dopaminergic vs. non-dopaminergic agents) and DBS. It highlights the complexity and significance of managing these conditions to enhance the quality of life for individuals with Parkinson's disease.

While these articles present notable contributions, areas for enhancement and critique can be discerned. The current landscape of movement disorder therapeutics, while holding promise, grapples with challenges such as the standardisation of treatment protocols, assessment of long-term efficacy, and the adoption of personalised medicine approaches. Some articles are lacking in their exploration of the translational implications of their discoveries, potentially limiting their practical impact within clinical settings. Rigorous clinical trials and comparative studies are deemed indispensable to validate the efficacy and safety of emerging therapeutic modes for their seamless integration into conventional treatments. Furthermore, factors like the elevated costs associated with novel therapies, the requisite for specialised training, and the accessibility of advanced treatments stand as significant hurdles necessitating attention [12]. Shortcomings in awareness, regulatory obstacles, cultural factors, and uneven healthcare distribution impede the adoption of new interventions worldwide [13].

The articles showcased in this special issue provide valuable insights into various movement disorders and their management, illustrating the continuous evolution of the field of movement disorder therapeutics with a wide variability demonstrated across different global regions [14]. To shape the future of movement disorder treatment and uplift patient outcomes and well-being, addressing identified areas for improvement and incorporating emerging technologies and collaborative care models are pivotal steps towards advancing the management of these movement disorder conditions.

## Declaration of competing interest

The authors declare that they have no known competing financial interests or personal relationships that could have appeared to influence the work reported in this paper.
